# TH1177, Clemastine, and Benzhydryl Derivatives Act as Novel Extracellular Blockers of ORAI Channels

**DOI:** 10.3390/biom16091372

**Published:** 2026-09-21

**Authors:** Pengyun Li, Hussein N. Rubaiy, Thomas Hallett, Yi Liang, Gui-Lan Chen, Bo Zeng, Pingping Zheng, Nawel Zaibi, Dong-Na Su, Andrew N. Boa, Shang-Zhong Xu

**Affiliations:** 1 Centre for Biomedicine, Hull York Medical School, University of Hull, Hull HU6 7RX, UK; lpyun@swmu.edu.cn (P.L.); hussein.rubaiy@ki.se (H.N.R.); thallett@live.co.uk (T.H.); chenguilan@swmu.edu.cn (G.-L.C.); zengbo@swmu.edu.cn (B.Z.); pingpingzh0615@gmail.com (P.Z.); nzaibi@outlook.com (N.Z.); sdn1000@21cn.com (D.-N.S.); 2 Key Laboratory of Medical Electrophysiology, Ministry of Education and Electrophysiological Key Laboratory of Sichuan Province, Collaborative Innovation Center for Prevention of Cardiovascular Diseases, Institute of Cardiovascular Research, Southwest Medical University, Luzhou 646000, China; liangyilz@163.com; 3 Department of Chemistry, School of Natural Science, University of Hull, Hull HU6 7RX, UK; a.n.boa@hull.ac.uk

**Keywords:** calcium channels, ORAI channels, stromal interaction molecule 1, TH1177, clemastine, cloperastine

## Abstract

ORAI channels are store-operated Ca^2+^ channels, which play important roles in physiology and pathophysiology. ORAI channel modulators are useful for developing pharmacological interventions. Here, we aimed to investigate the benzhydryl-derived analogues as novel ORAI channel blockers. The effects of benzhydryl-derived analogues TH1177, clemastine, cloperastine, and diphenhydramine were investigated in HEK-293 cells overexpressing individual ORAI isoforms and STIM1. The ORAI currents were recorded by patch-clamp, and cytosolic Ca^2+^ was measured by a fluorescent Ca^2+^ dye. TH1177 inhibited ORAI1, ORAI2, and ORAI3 currents with IC_50_ values of 18.3 μM, 4.8 μM, and 35.6 μM, respectively. Clemastine inhibited ORAI1 and ORAI2, but showed no effect on ORAI3. Both cloperastine and diphenhydramine, without a pyrrolidine ring structure, only showed minimal inhibition of ORAI1. Intracellular application of TH1177 and outside-out patch demonstrated that TH1177 blocked ORAI3 currents via the extracellular surface. TH1177 did not affect cytosolic STIM1 clustering and movement. Moreover, TH1177 potently inhibited cell proliferation with IC_50_ values of 2.6 µM for HAEC, 5.2 µM for EA.hy926, and 29.4 µM for HK-2 cells and induced apoptotic cell death. It is concluded that TH1177 is a pan-inhibitor for ORAI1-3 channels, while clemastine inhibits ORAI1 and ORAI2 but shows little effect on ORAI3. The pyrrolidine ring structure in the compounds of TH1177 and clemastine is critical for their inhibitory activity. The two benzhydryl derivatives (cloperastine and diphenhydramine) without the pyrrolidine ring only showed minimal inhibition of ORAI1. Our findings give a new pharmacological profile of ORAI channels.

## 1. Introduction

Ca^2+^-permeable ORAI channels reside within the plasma membrane and represent the defining molecular counterparts of Ca^2+^ release-activated Ca^2+^ (CRAC) channels [1,2]. Signals originating from activated G protein-coupled receptors or protein-tyrosine-kinase-coupled receptors drive the loss of Ca^2+^ from endoplasmic reticulum stores. Store depletion subsequently promotes cytosolic STIM1 clustering and subplasmalemmal translocation, which is evoked by the activation of inositol 1,4,5-trisphosphate receptors (IP_3_Rs) or intracellular ryanodine receptors [3]. There are three isoforms of ORAI channels, i.e., ORAI1, ORAI2, and ORAI3, which are ubiquitously expressed and abundant in immune cells, vasculature, kidney, and pancreas [4]. ORAI channels play critical roles in human physiology and disease processes; for example, mutation of ORAI1 (R91W) with loss-of-function leads to immune deficiency [1]; knockout of Orai1 retards the pathological formation of thrombus [5]; inhibition of ORAI channels impairs albumin uptake in proximal tubules and enhances diabetic albuminuria [3]; pharmacological inhibition of the ORAI1 channel by CM5480 prevents progression of chronic pancreatitis [6] and cytokine storms in patients with COVID-19 [7]. Moreover, ORAI and STIM proteins are critical targets in cancer biology through regulating cell proliferation and apoptosis [8]. Thus, ORAI channels can provide new therapeutic targets for intervention in immune-related dysfunction, cardiovascular diseases, diabetic complications, inflammation, and multiple types of cancer. Searching for ORAI channel-specific small molecules or pharmacological tools is therefore essential to understand the roles of ORAI channels in disease pathogenesis and for the therapeutic development of pharmacological intervention.

Specific ORAI channel modulators are still very limited, although some compounds have been reported by us and others [3,9,10,11]. We have characterized several ORAI channel pan-inhibitors, such as store-operated channel blockers BTP2 (also named YM-58483) and diethylstilbestrol (DES) [3], 4-chloro-3-ethylphenol and its analogues [12], and mibefradil [9]. The GSK-7975A analogues [10,13], CalciMedica compounds [6,14], and Synta66 [15] have also been reported. However, specific inhibitors for ORAI channels are still lacking, particularly ORAI isoform-specific modulators, which are largely unknown.

In this study, we evaluated how benzhydryl-derived analogues modulate ORAI channels in HEK-293 T-REx cells inducibly expressing ORAI isoforms and STIM1-EYFP. We have identified that TH1177 and clemastine are novel inhibitors of ORAI channels, and clemastine is more specific for ORAI1 and ORAI2 without affecting ORAI3. The pyrrolidine ring structure in the analogues is critical for the inhibitory activity on ORAI channels.

## 2. Materials and Methods

### 2.1. Cell Culture and Transfection

HEK-293 T-REx cells inducibly overexpressing ORAI genes were used, and the details are described previously in our reports [9]. Briefly, human ORAI sequences (GenBank accession numbers: ORAI1 (NM_032790), ORAI2 (BC069270), and ORAI3 (NM_152288)) were inserted into the tetracycline-inducible expression system pcDNA4/TO. ORAI1- and ORAI2-coding constructs were tagged with mCherry (mCherry-ORAI1 and mCherry-ORAI2), whereas ORAI3 was tagged with monomeric cyan fluorescent protein (mCFP) (mCFP-ORAI3). These plasmids were transfected into the T-REx™-293 cell line (RRID: CVCL_D585) (Invitrogen, Paisley, UK), followed by stable co-expression of STIM1-EYFP alongside ORAI isoforms. The gene expression of ORAIs was induced by tetracycline (1 µg∙mL^−1^) for 1–3 days before patch recording or Ca^2+^ imaging. HEK-293 T-REx cells without gene transfection or cells without addition of tetracycline were set as controls. HEK-293 T-REx cells were grown in DMEM/F-12 GlutaMAX™ medium (Invitrogen, UK) supplemented with 10% fetal calf serum (FCS), penicillin (100 units∙mL^−1^), and streptomycin (100 µg∙mL^−1^). Cells were cultured at 37 °C with 95% air plus 5% CO_2_ and seeded onto glass coverslips for functional recordings.

Human aortic endothelial cells (HAECs) were obtained from PromoCell (Cat. No. C-12271, Heidelberg, Germany). EA.hy926, an immortal vascular endothelial cell line originating from human umbilical vein endothelial cells (HUVECs), was obtained from American Type Culture Collection (ATCC) (RRID: CVCL_3901, Cat. No. CRL-2922, Middlesex, UK). Endothelial cell cultures were maintained in an endothelial cell medium (PromoCell, Heidelberg, Germany, Cat. No. C-22110) containing 2% fetal calf serum (FCS), recombinant human epidermal growth factor (0.1 ng/mL), and basic fibroblast growth factor (1 ng∙mL^−1^). HAECs were utilized at passages 2–3 in the study. The human proximal tubular cell line (HK-2) was obtained from LGC Standards (Teddington, UK, RRID: CVCL_0302, Cat. No. CRL-2190).). HK-2 cells were cultured in DMEM/F-12 medium containing 5.5 mM glucose, enriched with 10% FCS, 10 mM HEPES, and penicillin and streptomycin antibiotics, as described above. The immortalized human podocytes were obtained from Prof. M. Saleen (Bristol University) and maintained at 33 °C in RPMI 1640 medium containing 10% FBS and 1% insulin-transferrin-selenium ((ITS-G, Gibco, ThermoFisher Scientific, Paisley, UK. Cat. No. 41400045). All cells were confirmed to be contamination-free by mycoplasma testing. All cells were cultured in CO_2_ incubators (5% CO_2_).

### 2.2. Electrophysiology

The whole-cell patch-clamp recordings were conducted following protocols reported in our previous report [3,9]. Briefly, electrical signals were amplified with an Axopatch 200B amplifier, and data acquisition was controlled via pClamp software (version 10). A 1000 ms ramp voltage stimulus ranging from –100 mV to +100 mV was delivered at a frequency of 0.2 Hz. Electrical signals were digitized and sampled at 3 kHz and low-pass-filtered at 1 kHz. The glass microelectrodes were pulled using a two-stage puller (PC-10, Narishige, Tokyo, Japan), and electrodes with a tip resistance between 3 and 5 MΩ were selected for patch recording. The intracellular pipette solution contained the following (in mM): 145 Cs-methanesulfonate, 8 MgCl_2_, 10 BAPTA, and 10 HEPES. The pH was adjusted to 7.2 by addition of 1 M CsOH. The same intracellular solution was also applied for outside-out patch recordings. The standard bath solution comprised the following (in mM): 130 NaCl, 5 KCl, 1.5 CaCl_2_, 10 HEPES, 1.2 MgCl_2_, and 8 D-glucose, and its pH was adjusted to 7.4 using 2 M NaOH. The same recordings were additionally obtained using a divalent cation-free solution that contained the following (mM): 150 NaCl, 10 EDTA, 10 tetraethylammonium chloride, 10 HEPES, and 10 D-glucose. All electrophysiological experiments were carried out at room temperature. For dose–response curves, current amplitudes were normalized to the stable baseline current recorded before compound application. Data were fitted to a standard Hill equation to derive IC_50_ values using OriginPro software version 10 (OriginLab, Northampton, MA, USA). All fitting parameters were kept consistent across ORAI isoforms.

### 2.3. Live Cell Ca^2+^ and Fluorescence Imaging

Cells were pre-incubated with 2 µM Fura-PE3/AM Ca^2+^ dye at 37 °C for 30 min. The loading buffer was a Ca^2+^-free bath solution containing the following (in mM): 130 NaCl, 5 KCl, 1.2 MgCl_2_, 10 HEPES, 8 D-glucose, and 0.4 EGTA. Following incubation, cells were washed twice using standard extracellular bath solution. The Ca^2+^ fluorescence ratio was dynamically sampled at wavelengths of 340 nm and 380 nm (F_340_/F_380_) with a FlexStation 3 microplate reader (Molecular Devices, San Jose, CA, USA). The control samples were set on the identical 96-well culture plate and measured in parallel. To monitor STIM1-EGFP translocation, detailed methods are described in our previous report [3,9]. Briefly, stably transfected STIM1-EYFP cells were plated onto glass coverslips and cultured for 1–2 days. Live cell fluorescence signals for STIM1-EYFP were visualized using an inverted Nikon fluorescence microscope with a high-magnification oil objective (Plan Fluor 100×/1.30) and software (NIS-Elements Version 3.2, Nikon, Tokyo, Japan). STIM1 puncta were defined using a 2-fold intensity threshold relative to background fluorescence. All fluorescence-imaging assays were carried out at room temperature.

### 2.4. Cell Proliferation and Viability Assay

Cell proliferation was assessed using a water-soluble tetrazolium-1 (WST-1) colorimetric assay (Roche Applied Science, Basel, Switzerland. Cat. no. 11644807001). In this assay, mitochondrial dehydrogenase enzymes within viable cells cleave tetrazolium salts to yield formazan products. The production of formazan is positively correlated with the number of proliferative cells, allowing reliable evaluation of cell growth. Meanwhile, extracellular lactate dehydrogenase (LDH) release in the culture medium was detected to assess cellular cytotoxicity (Roche Applied Science, cat. no. 11644793001). The WST-1 proliferation assay and LDH cytotoxicity assay were performed according to protocols described in our previous report [9]. Briefly, cells were seeded into 96-well plates at a density of 4 × 10^3^ cells/well and cultured overnight prior to compound treatment. Cells were incubated with a series of concentrations of test compounds for 48 h. WST-1 reagent was added according to the manufacturer’s protocol, followed by 2 h incubation at 37 °C before absorbance measurement at 490 nm. For LDH assays, cell-free culture supernatants were collected after 48 h of compound incubation, and LDH reactions were developed as instructed. Absorbance was read at 450 nm using a microplate spectrophotometer. All absorbance values were background-corrected against blank controls. Quantitative analysis of absorbance signals generated by WST-1 and LDH assays was achieved using a spectrophotometer.

### 2.5. Flow-Cytometric Assessment of Cell Cycle Profiles

Propidium iodide (PI) staining coupled with fluorescence-activated cell sorting (FACS) was used to investigate TH1177-mediated cell cycle alterations. Briefly, HK-2 cells were seeded into 6 cm culture dishes at 5.0 × 10^3^ cells/mL and allowed to attach for 24 h, followed by treatment with TH1177 (1, 5, and 25 μM) for an additional 24 h. Cells were then collected by trypsin–EDTA (0.25%) digestion, rinsed twice with ice-cold phosphate-buffered saline (PBS), and fixed overnight at 4 °C in 70% ice-cold ethanol. Following fixation, residual ethanol was removed by PBS washing, and cellular RNA was degraded via 30 min incubation with RNase A (100 μg/mL) at 37 °C. Cells were then incubated with PI working solution (50 μg/mL PI, 0.1% Triton X-100 in PBS) at 4 °C for 30 min under dark conditions. Labelled cells were analyzed using a BD flow cytometer. PI fluorescence was excited at 488 nm, and emission signals were collected at 617 nm. Data acquisition was performed at room temperature. No fewer than 10,000 single-cell events were acquired per sample. Cell cycle distribution (G_0_/G_1_, S, and G_2_/M phases) and the sub-G_1_ apoptotic fraction were quantified using Summit 4.0 software. Each independent experiment included technical triplicates, and experiments were repeated for at least three independent biological replicates.

### 2.6. Chemicals

Clemastine fumarate, cloperastine, diphenhydramine, thapsigargin (TG), 2-aminoethoxydiphenyl borate (2-APB), and tetracycline hydrochloride were ordered from Sigma-Aldrich. BTP2 was obtained from R&D Systems (Abingdon, UK). TH1177 was obtained from Prof. Michael Shattock (King’s College London) with a research-grade purity of >95%. Fura-PE3/AM was obtained from Thermofisher Scientific (UK). The stock solutions of Fura-PE3/AM (5 mM) and 2-APB (100 mM) were prepared by dissolving the powders in 100% dimethyl sulfoxide. All common salts employed in this work were obtained from Sigma-Aldrich (Gillingham, UK).

### 2.7. Statistics

Data are presented as mean ± SEM. The sample size *n* corresponds to the number of individual cells analyzed in patch-clamp recordings and the number of assay wells for calcium measurement using FlexStation 3. Paired Student’s *t*-test was applied to compare values obtained before and after compound treatment. For comparisons across three or more experimental groups, one-way ANOVA followed by Bonferroni’s post hoc analysis was performed. Statistical significance was assumed at *p*-values less than 0.05.

## 3. Results

### 3.1. Effects of Benzhydryl-Derived Analogues TH1177, Clemastine, Cloperastine, and Diphenhydramine on ORAI Channels

**Figure 1:**
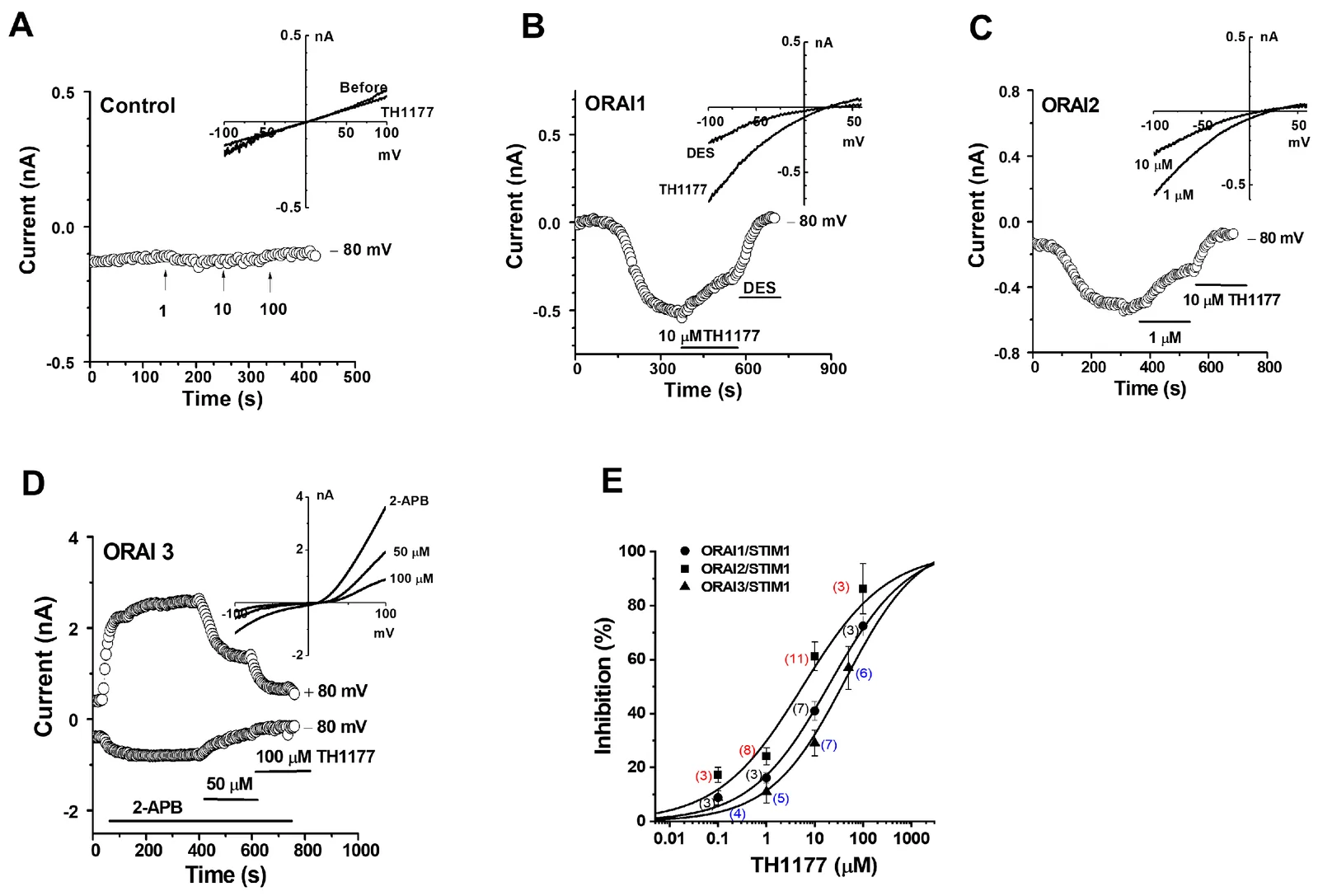
TH1177 inhibits ORAI channels. (**A**) Whole-cell current recorded in control cells (control) without addition of tetracycline to induce gene expression. (**B**) ORAI1 current recorded from cells overexpressing mCherry-ORAI1/STIM1-EYFP. Currents were activated by TG (1 µM) in the pipette solution, followed by sequential application of TH1177 (10 µM) and the ORAI channel blocker diethylstilbestrol (DES, 10 µM). (**C**) ORAI2 current from cells overexpressing mCherry-ORAI2/STIM1-EYFP. TG (1 μM) was used to trigger currents, which were then exposed to TH1177 at 1 μM and 10 μM. (**D**) ORAI3 current in cells transfected with CFP-ORAI3/STIM1-EYFP was evoked by 2-APB (100 µM), after which TH1177 (50 μM, 100 μM) was administered. (**E**) Dose–response curves illustrating the inhibitory effects of TH1177 on ORAI1, ORAI2, and ORAI3 channels, with IC_50_ values of 18.3 µM, 4.8 µM, and 35.6 µM, respectively. Sample sizes (*n*) are indicated in parentheses.

The current was recorded by whole-cell patch-clamp in the HEK-293 T-REx cells co-expressing human ORAI1-3 tagged with mCFP or mCherry and STIM1-EYFP. The expression of ORAI channels was induced by tetracycline treatment and verified by detection of their tagged fluorescent reporter proteins in plasma membrane, as we described previously [3]. The ORAI1 and ORAI2 currents were evoked by 1 μM TG, while the ORAI3 currents were activated using 2-APB (100 µM), a direct channel activator of ORAI3 [16]. The TG-evoked ORAI1 and ORAI2 currents reached a maximum in ~5 min after the membrane breakthrough of the whole-cell patch, while the current of ORAI3 showed a robust current with fast activation and a typical IV curve shape with double (inward and outward) rectifications after superfusion with 2-APB (Figure 1). No notable TG-evoked currents were detected in the non-induced HEK-293 T-REx cells, which are shown as negative controls (Figure 1A). Using such cell models overexpressing specific targets, we screened a series of small molecules and found that TH1177 inhibited ORAI1, ORAI2, and ORAI3 potently (Figure 1B–D). TH1177 inhibited ORAI currents in a concentration-dependent manner, with IC_50_ values of 18.3 µM, 4.8 µM, and 35.6 µM for ORAI1, ORAI2, and ORAI3, respectively (Figure 1E), suggesting that TH1177 is a pan-inhibitor for ORAI channels, but seems to be more potent for the ORAI2 isoform.

**Figure 2:**
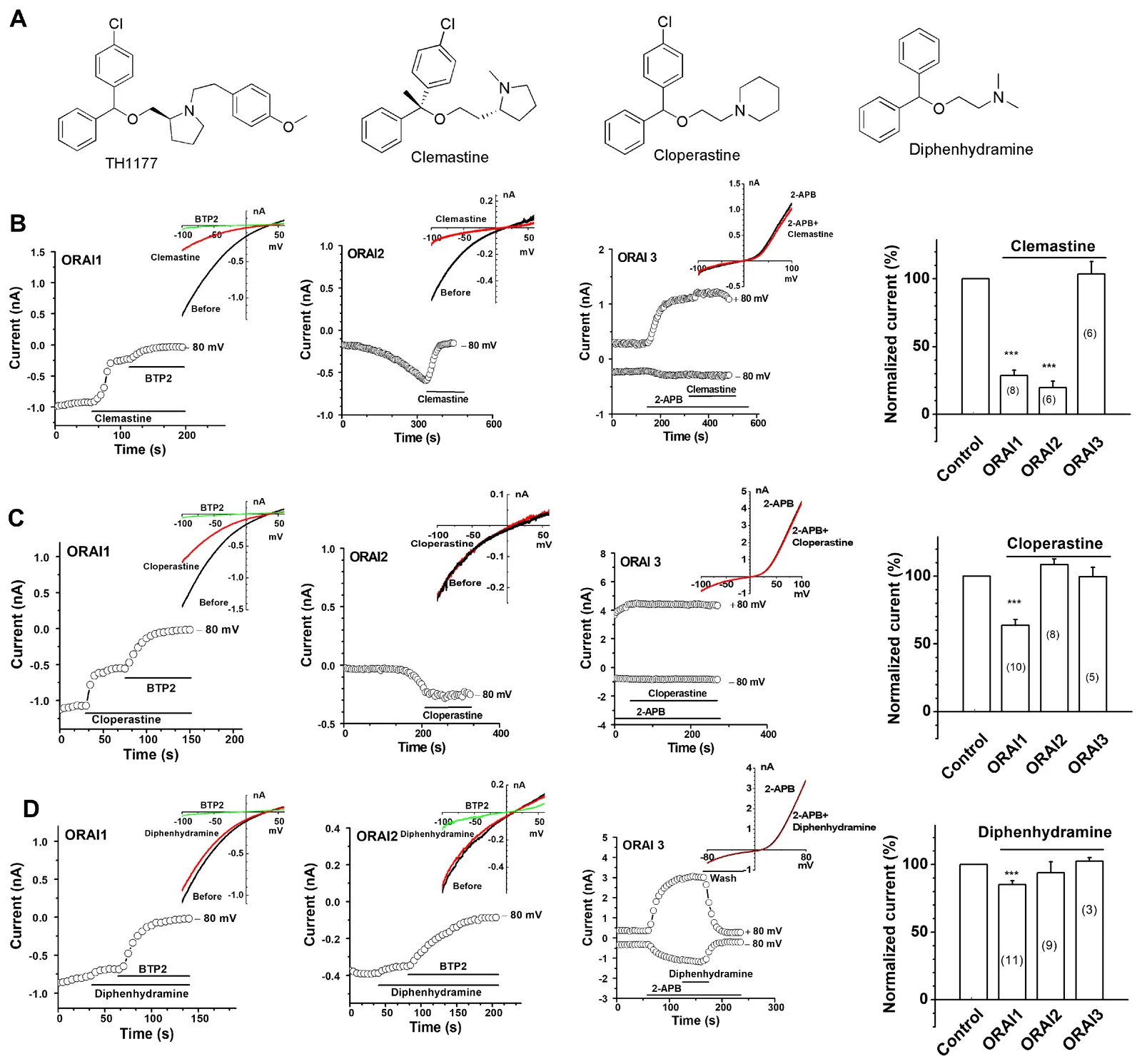
Effects of benzhydryl derivatives on the currents of ORAI channels. (**A**) Structure of benzhydryl derivatives. (**B**) Effect of clemastine (100 µM) on the currents of ORAI1-3. (**C**) Effects of cloperastine (100 µM). (**D**) Effects of diphenhydramine (100 µM). BTP2 (10 µM), a pan-blocker, was used as a positive control in some recordings (green trace in the insets). The normalized mean data are shown in the bar chart (*n* numbers are shown in parentheses for each group, *** *p* < 0.001).

We also tested the benzhydryl-derived analogues clemastine, cloperastine, and diphenhydramine, which have a structural similarity to TH1177 (Figure 2A). The three compounds are H1-antihistamine drugs. Interestingly, clemastine inhibited both ORAI1 and ORAI2 currents, but exerted no inhibitory effect on ORAI3 (Figure 2B, Supplementary Figure S1). The IC_50_ of clemastine for ORAI1 was 68.6 µM (Supplementary Figure S2). However, cloperastine and diphenhydramine, with a structure similar to TH1177 but without the pyrrolidine residues, slightly inhibited ORAI1 current but had no effect on ORAI2 and ORAI3 currents (Figure 2C,D), suggesting that the pyrrolidine ring is critical for channel-blocking activity.

### 3.2. Extracellular Inhibition of ORAI Channels by TH1177

**Figure 3:**
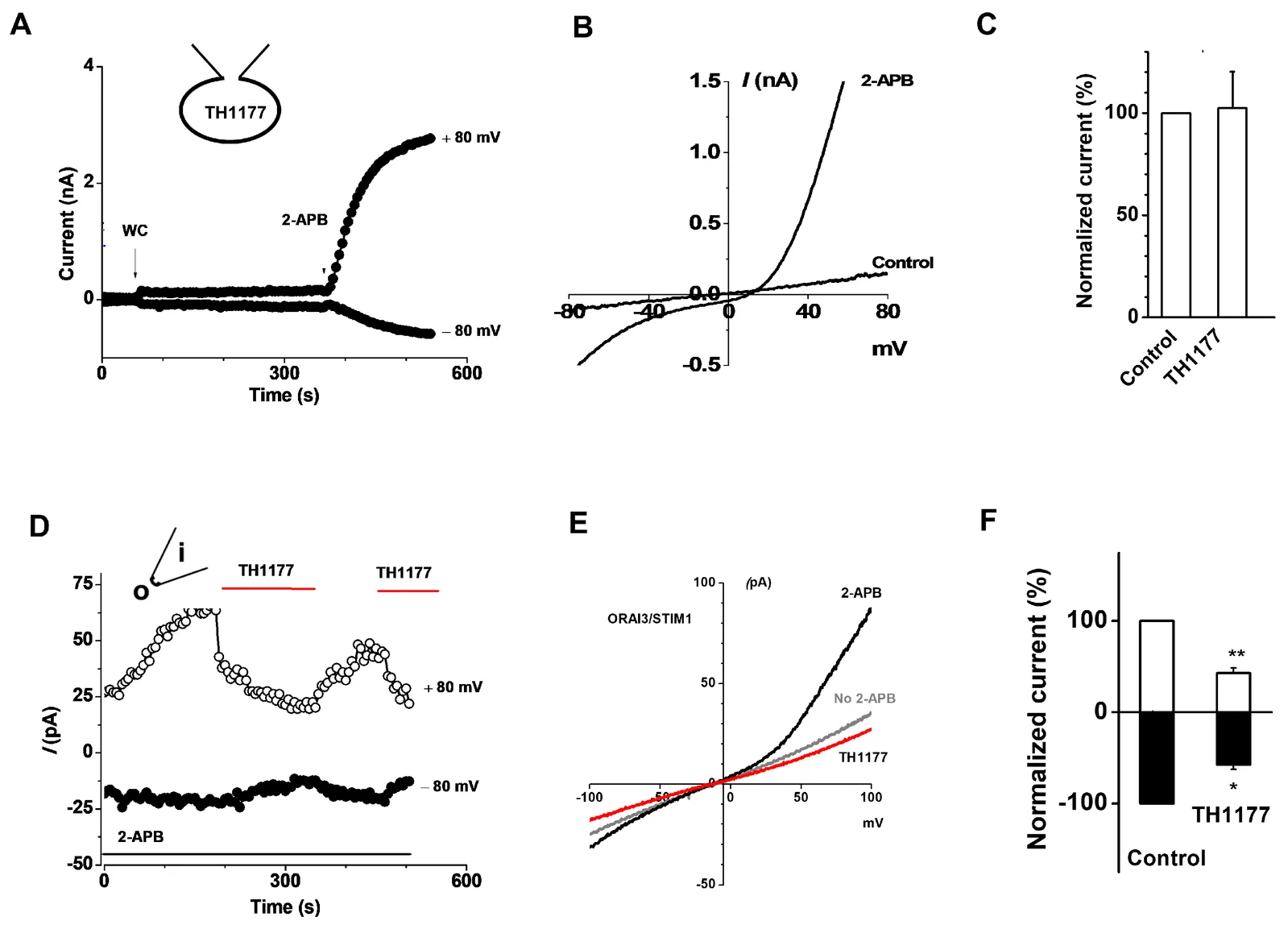
Extracellular effect of TH1177 on ORAI3 channels. (**A**) Whole-cell current was recorded from ORAI3-expressing cells, where 100 µM TH1177 was included in the pipette solution, and 100 µM 2-APB was superfused via bath solution. (**B**) I–V curve for (**A**). (**C**) Summarized data for 2-APB-evoked currents obtained with or without 100 µM TH1177 (control) in the pipette solution (*n* = 5 per group). (**D**) Representative outside-out patch traces illustrating the inhibitory effect of 10 µM TH1177. (**E**) I–V curve for the outside-out patch recording shown in (**D**). (**F**) Normalized data for TH1177-suppressed currents in outside-out patches (*n* = 5 per group, * *p* < 0.05, ** *p* < 0.01).

The extracellular effect of TH1177 was investigated on ORAI3. The concentration of TH1177 (100 µM) was added to the pipette solution and diffused into cells to determine whether intracellular TH1177 could prevent ORAI3 channel activation stimulated by 2-APB. After a 5 min equilibration period following whole-cell breakthrough, the ORAI3 activator 2-APB at 100 µM was superfused using bath solution. It was found that internal application of TH1177 was unable to prevent ORAI3 channel activation by the activator 2-APB (Figure 3A–C). This finding suggests that TH1177 exerts its inhibitory effect on ORAI3 at the extracellular side of this transmembrane protein.

To further verify this extracellular site of action, the outside-out patch-clamp was carried out on the cells co-expressing ORAI3/STIM1. TH1177 at 10 µM reduced the amplitude of ORAI3 current significantly in the outside-out patch recordings, and the inhibitory effect was reversible by washout (Figure 3D–F).

### 3.3. Effect of TH1177 on Cytosolic STIM1 Movement

**Figure 4:**
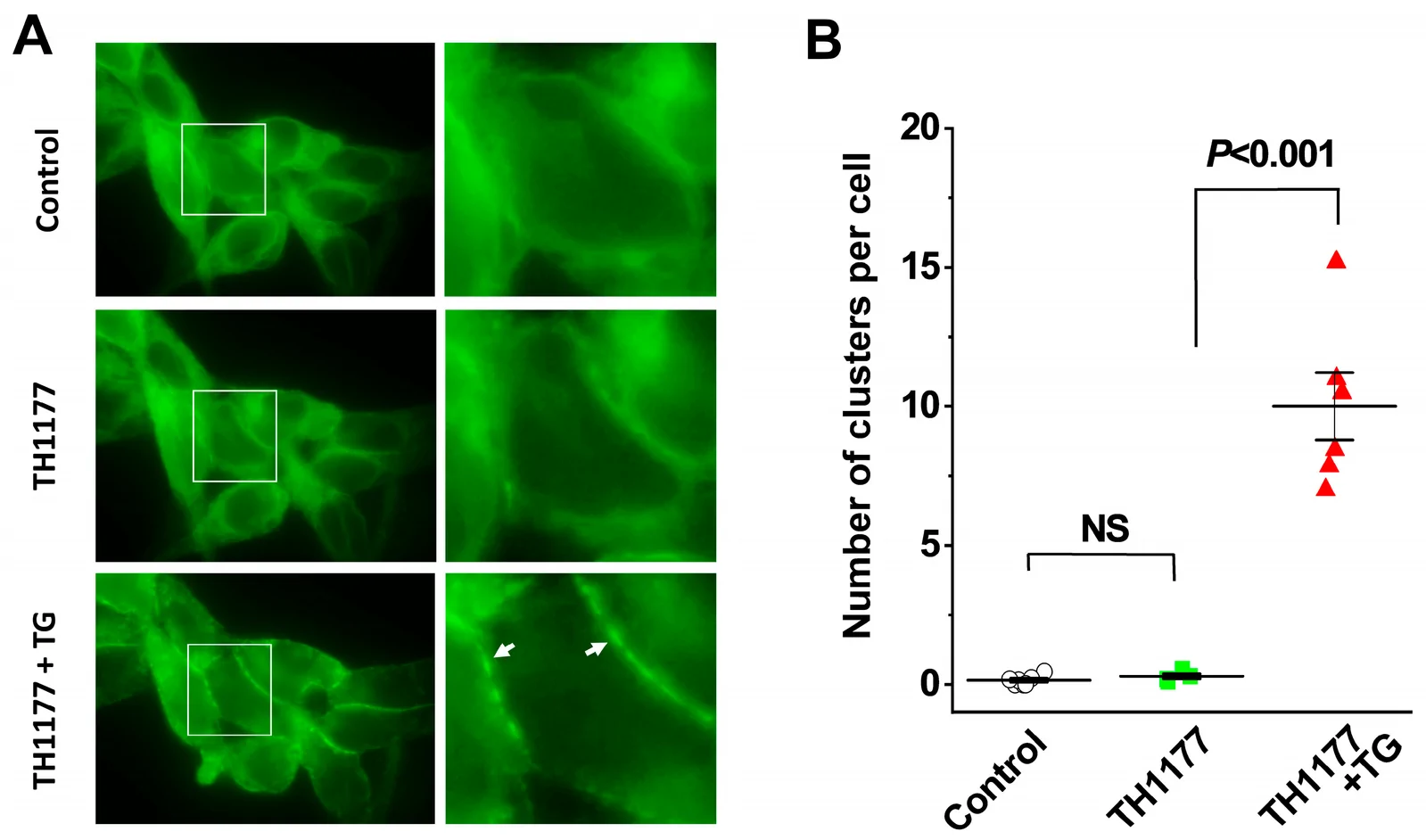
Ca^2+^ store depletion-induced STIM1 subplasmalemmal translocation and clustering and the inhibitory effect of TH1177 in stably STIM1-EYFP-transfected cells. (**A**) Example of STIM1 cluster (puncta) formation at the plasma membrane. Magnified views correspond to the boxed regions in the left panels. The arrows indicate the subplasmalemmal STIM1 puncta. Cells were pre-incubated with TH1177 (50 μM) for 10 min prior to 1 μM TG application (TG + TH1177), while cells treated with TH1177 alone and untreated cells served as the other two groups. (**B**) Quantitative summary of STIM1 puncta per cell is pooled from 4 to 7 independent experiments. The total cell numbers are 89, 47, and 55 for control, TH1177 (50 µM), and TG (1 µM) + TH1177 (50 µM) groups, respectively. NS, not significant.

The clustering and movement of STIM1 from the ER to the subplasmalemmal area in the cytosol is an essential step for ORAI store-operated channel activation. The coupling of ORAI and STIM1 triggers channel opening and store-operated Ca^2+^ influx. STIM1 movement can serve as a target for ORAI channel regulators [9,17]. We therefore examined the effect of TH1177 on STIM1 movement. In STIM1-EYFP stable HEK-293 cells, subplasmalemmal STIM1 translocation and clustering were induced by TG, a sarco/endoplasmic reticulum Ca^2+^-ATPase (SERCA) inhibitor used to deplete ER Ca^2+^ stores. In the cells pretreated with TH1177, STIM1-EYFP clustering was also evident near the plasma membrane (Figure 4). This result indicated that TH1177 did not change STIM1 clustering and intracellular translocation.

### 3.4. Effects of TH1177 on Ca^2+^ Release and SOCE

**Figure 5:**
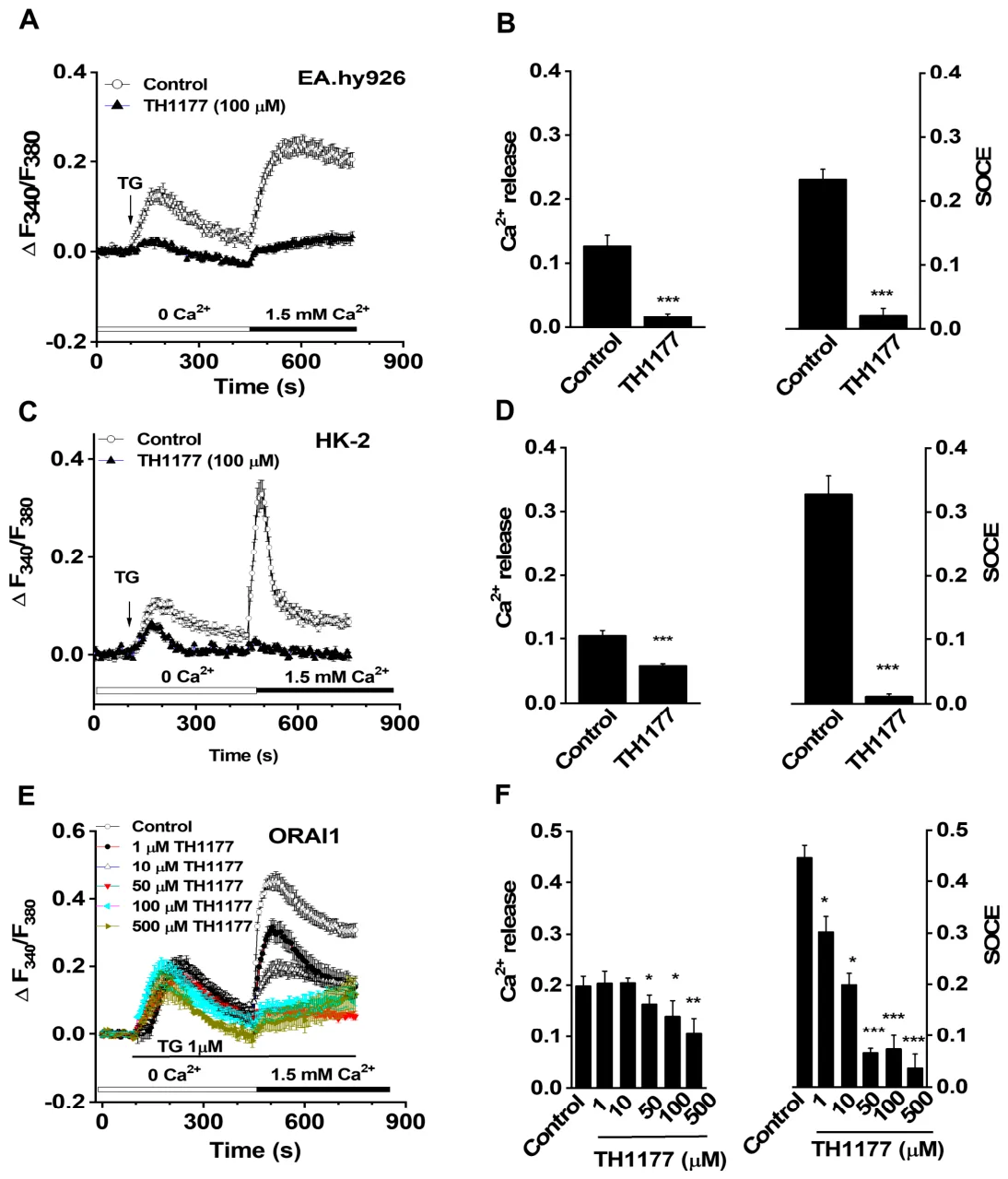
ER Ca^2+^ release and SOCE inhibition by TH1177. (**A**) Fura-PE3/AM-loaded EA.hy926 endothelial cells were stimulated with 1 μM thapsigargin (TG) to initiate ER Ca^2+^ mobilization. Changes in the F_340_/F_380_ fluorescence ratio were tracked for TH1177-pretreated and control groups. The SOCE was elicited by re-addition of 1.5 mM Ca^2+^ into the bath solution. (**B**) Quantitative analysis of the peak amplitudes of Ca^2+^ release or SOCE (*n* = 8). (**C**) Representative traces of ER Ca^2+^ release and SOCE recorded in HK-2 cells. (**D**) Mean data obtained from HK-2 cells (*n* = 8). (**E**) Concentration-dependent effects of TH1177 on ER Ca^2+^ release and SOCE in HEK-293 T-REx cells expressing ORAI1. (**F**) Mean data for (E) (*n* = 8, * *p*  <  0.05, ** *p*  <  0.01, *** *p*  <  0.001).

We examined TH1177-dependent changes in ER Ca^2+^ release using EA.hy926 endothelial cells, the human proximal tubular cell line HK-2, and HEK-293 T-REx cells. TG stimulation elicited substantial Ca^2+^ release from the ER. The peak amplitude of the TG-induced Ca^2+^ release transient was inhibited by a high concentration (100 µM) of TH1177 in EA.hy926 cells (Figure 5A,B) and HK-2 cells (Figure 5C,D). The concentration-dependent inhibition of Ca^2+^ release was also observed in T-REx ORAI1 (Figure 5E,F), ORAI2, and ORAI3 cells (Supplementary Figure S3). Lower TH1177 concentrations (1–10 µM) showed no significant effect on ER Ca^2+^ release but significantly inhibited ORAI1-mediated SOCE with an IC_50_ of 4.57 ± 3.03 µM (Figure 5F), suggesting high sensitivity of TH1177 to ORAI1 SOCE. For ORAI2-expressing cells (Supplementary Figure S3), TH1177 at 500 µM significantly suppressed ER Ca^2+^ release, whereas SOCE was markedly inhibited at concentrations of 50 µM or more. In ORAI3-expressing cells, TH1177 at both 50 µM and 500 µM reduced Ca^2+^ release, and SOCE was significantly inhibited at 100 µM. Collectively, these results indicate that while ORAI1-mediated SOCE is the most sensitive target of TH1177, higher concentrations are required to inhibit SOCE in ORAI2 and ORAI3 channels, and ER Ca^2+^ release is only affected at much higher concentrations. Knockdown of ORAI1 with specific siRNAs significantly suppressed SOCE, but failed to alter Ca^2+^ release, as we demonstrated previously [3,9]. This suggests that the inhibition of ER Ca^2+^ release by higher concentrations of TH1177 is a non-specific effect and unrelated to the direct block of ORAI channels.

### 3.5. Effects of TH1177 on Cell Growth and Cell Death

**Figure 6:**
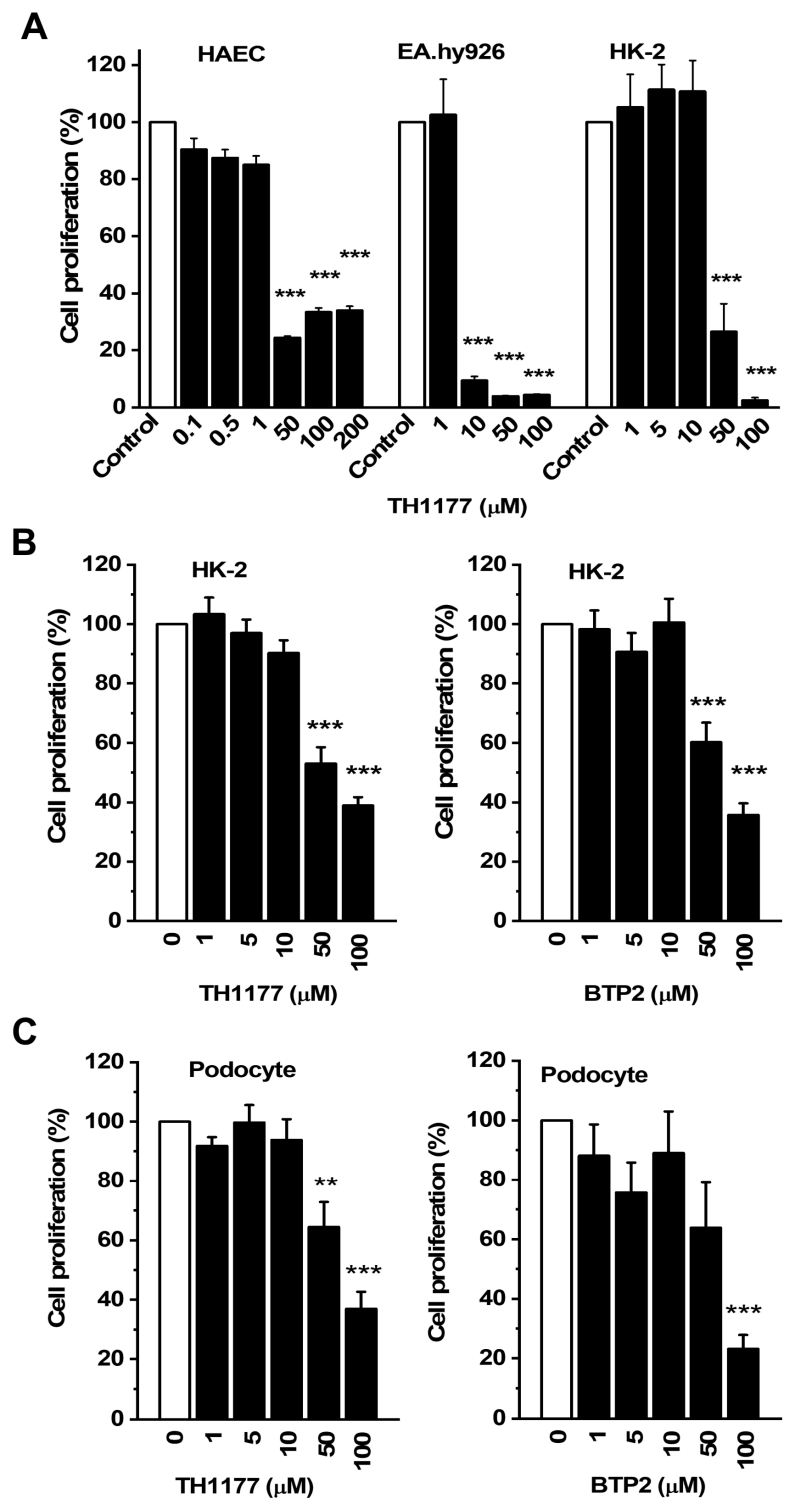
TH1177 regulates cell proliferation in multiple cell lines. (**A**) WST-1 assay was performed to evaluate the proliferation of HAEC, EA.hy926 cells, and HK-2 cells after TH1177 intervention. The absorbance was measured at 490 nm, with 650 nm set as the reference wavelength. (**B**) Comparative assessment of proliferative inhibition by TH1177 and BTP2 in HK-2 cells and (**C**) human podocytes. (*n* = 8 per group, ** *p* <  0. 01, *** *p*  <  0.001).

**Figure 7:**
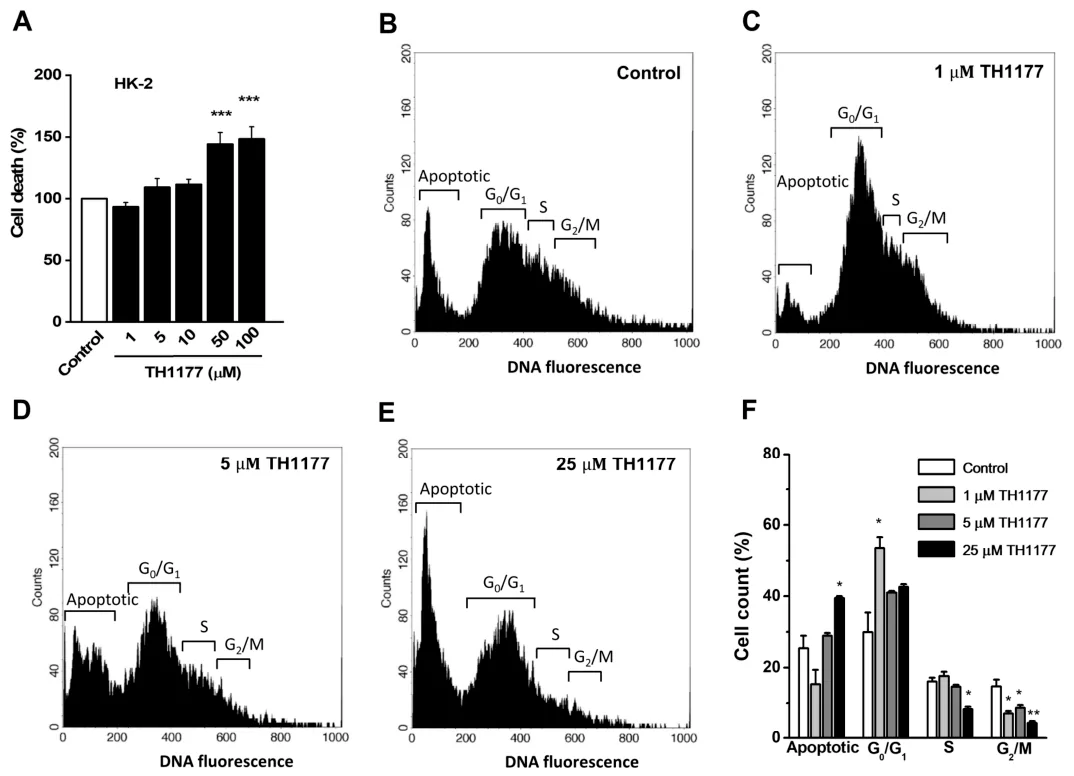
TH1177 modulates cell death in HK-2 cells. (**A**) The viability of TH1177-treated HK-2 cells was evaluated using an LDH cytotoxicity assay. (**B**–**E**) FACS was applied to analyze cell cycle distribution and cell death in HK-2 cells. Representative PI fluorescence histograms illustrate cell status in the vehicle control group (**B**) and groups treated with 1 μM (**C**), 5 μM (**D**), and 25 μM (**E**) TH1177. (**F**) Data are presented as the mean ± SEM from three independent experiments (*n* = 6, * *p* < 0.05, ** *p* < 0.01, *** *p* < 0.001 compared with the control).

ORAI channels play critical roles in cancer cell proliferation and invasion, and the blockage of ORAI channels may have anti-proliferative and anti-tumour effects [2,18]. We therefore further explored whether TH1177 affects cell growth, cell death, and cell cycle distribution. TH1177 not only significantly inhibited the proliferation of HAEC, but also the proliferation of the immortalized endothelial cell line EA.hy926 and the proximal tubular cell line HK-2. The IC_50_ values for anti-proliferation by TH1177 for HAEC, EA.hy926, and HK-2 were 2.6 µM, 5.2 µM, and 29.4 µM, respectively (Figure 6A, Supplementary Figure S4), suggesting that the vascular endothelial cells are more sensitive to TH1177, while the HK-2 cells are slightly resistant. In the comparative experiments with the pan-inhibitor of ORAI channel BTP2, the inhibitory effects on HK-2 and podocyte proliferation were observed at concentrations higher than 50 µM, and both TH1177 and BTP2 showed similar potency on anti-proliferation (IC_50_ = 11.4 µM and 27.4 µM for TH1177 and BTP2 in HK-2; and 47.3 µM and 51.9 µM for podocyte, respectively) (Figure 6B,C). On the other hand, TH1177 at high concentrations (>50 µM) significantly resulted in cell death, as measured by LDH assay (Figure 7A). We also examined the effect on the cell cycle using flow cytometry (Figure 7B–F). Quantitative analysis confirmed that TH1177 at lower concentrations (1 μM and 5 μM) increased the percentage of cells in the G_0_/G_1_ phase, while high-concentration TH1177 (25 μM) induced apoptosis and decreased the percentage of cells in the S and G_2_/M phases. These results suggested that TH1177 can retard G_1_ progression in the cell cycle of HK-2 cells to S and G_2_/M phases.

## 4. Discussion

In the present study, we evaluated the effects of the benzhydryl derivatives TH1177, clemastine, cloperastine, and diphenhydramine on ORAI channel activity. TH1177 inhibited ORAI1, ORAI2, and ORAI3, whereas clemastine inhibited ORAI1 and ORAI2 without measurably affecting ORAI3. Cloperastine and diphenhydramine produced a small inhibitory effect on ORAI1 and had little or no effect on ORAI2 and ORAI3. The inhibitory effect of TH1177 is concentration-dependent and reversible and, at least for ORAI3, acted on an extracellularly accessible site. TH1177, at high concentrations, reduced both SOCE and Ca^2+^ release from the ER in vascular endothelial and proximal tubular cells. TH1177 also significantly inhibited cell growth and increased apoptosis by retarding cell cycle progression from the G_0_/G_1_ phase to the S and G_2_/M phases. These findings identify ORAI channels as previously unrecognized pharmacological targets of TH1177 and demonstrate distinct ORAI isoform profiles among structurally related benzhydryl compounds.

ORAI channels constitute the main molecular components of SOCE in many cell types, including vascular endothelial cells and HK-2 [3,9,19]. TH1177 potently inhibited SOCE in the native cells (EA.hy926 and HK-2 cells) and HEK-293 T-REx cells with an IC_50_ of 4.57 µM. This potency is similar to that previously reported for inhibition of the Cav3.2 channel by TH1177 (IC_50_ = 2.8 µM), and substantially greater than its reported potency against TRPV5 and TRPV6, for which the IC_50_ values are 456 µM and 675 µM, respectively [20]. Nevertheless, direct comparisons among these values should be interpreted cautiously because they may have been obtained using different expression systems and experimental conditions. Furthermore, non-specific or off-target effects cannot be excluded when TH1177 is used at high concentrations, as is the case for many small-molecule ion-channel inhibitors.

Whole-cell patch-clamp recordings showed that TH1177 inhibits all three ORAI isoforms, with ORAI2 displaying the greatest apparent sensitivity to the compound. The IC_50_ for ORAI2 inhibition was 4.8 µM, supporting the identification of ORAI2 as a relatively sensitive target of TH1177. Experiments using excised membrane patches (outside-out patch) and intracellular application of TH1177 during whole-cell recordings indicated that inhibition of ORAI3 involves an extracellularly accessible site. Although these experiments do not identify a specific binding residue or domain, they suggest the presence of an extracellular drug-interaction site that may be useful for future structure–activity studies and inhibitor development. Blockage of SOCE or ORAI channels has been shown to exert anti-proliferative effects in multiple cancer cell types and normal cell lines [2,8]; therefore, the inhibition of cell proliferation by TH1177 could be explained, at least partly, as its blocking effects on ORAI channels, although the inhibitory effects on T-type Ca^2+^ channels could also partly account for its anti-proliferative effects in some types of cancer cells [2]. STIM1-dependent reciprocal functional crosstalk has been described between the L-type Ca^2+^ channel CaV1.2 and ORAI1 [21], but the cellular and functional communications between ORAI and T-type Ca^2+^ channels remain unknown. However, inhibition of either ORAI or T-type Ca^2+^ channels can result in a significant decrease in cell growth and promote apoptotic cell death [2,8]. TH1177 more potently inhibits proliferation in vascular endothelial cells (HAECs and EA.hy926 cells) than in kidney cells (podocytes and proximal tubular cells), which may be attributed to the relatively higher ORAI expression in vascular endothelial cells, as we described previously [9]. The effect of TH1177 on the cell cycle was also observed in this study. Treatment with TH1177 increased the percentage of cells at phase G_0_/G_1_ while decreasing the percentage of cells at phase G_2_/M, which suggests that cell cycle progression has been retarded from G_1_ to S, and G_2_ to phase M. This finding is consistent with reports on ovarian cancer cells [22], T-lymphoma cell lines, and Jurkat cells [23], as well as our previous work on HK-2 cells [9], which provides evidence of pharmacological intervention on intracellular Ca^2+^ signalling via ORAIs by regulating DNA synthesis from the G_1_ to S phase and mitotic transition from the G_2_ to M phase [24]. However, high concentrations of TH1177 may have non-specific cytotoxicity by inducing apoptosis and decreasing the proportion of cells in the G_0_/G_1_ phase. In contrast, genetic strategies for specific inhibition of Ca^2+^-permeable channels, such as siRNA-mediated knockdown, predominantly increase the cell population in the G_2_/M phase [25]. Recently, several studies have also reported contributions of ORAIs/STIMs in cell apoptosis using siRNA [2,26,27,28]; therefore, the potential anti-tumour action of TH1177 can be regarded as blockage of ORAI store-operated channels.

Ca^2+^ release from ER serves as an initial trigger for ORAI channel activation, which is achieved through STIM1 clustering and subsequent translocation to the plasma membrane. TH1177 shows no effects on ER Ca^2+^ release and cytosolic STIM1 clustering and translocation at lower concentrations (<10 µM), suggesting that the blockage by TH1177 directly targets the channel protein. TH1177 at high concentrations (>50 µM) inhibits Ca^2+^ release and causes apoptosis of HK-2 cells, which suggests that TH1177 at high concentrations may damage kidney function. The mechanism by which high concentrations of TH1177 inhibit ER Ca^2+^ release remains unknown. Potential mechanisms include altered activity of SERCA, inositol 1,4,5-trisphosphate receptors, or ryanodine receptors. The SOCE in HK-2 cells was nearly abolished by ORAI siRNAs, suggesting that ORAI channels are dominant molecules of SOCE in such cells [3]. It is unclear what the pharmacological effects of TH1177 on proteinuria in vivo are; however, it has been reported that there are no effects on proteinuria and blood pressure in the in vivo rat model with Thy1 nephritis; however, it seems to reduce glomerular injury and aberrant proliferation of mesangial cells [29]. We note that our findings are derived from this heterologous system; the in vivo effects have not been assessed in the current study, and future work employing endogenous gene-edited models is required for further validation. Any potential therapeutic benefits will also need to be confirmed in subsequent animal studies.

We have also evaluated several T-type channel blockers with various chemical structures, for example, mibefradil, ML218, and ethosuximide [9]. Both mibefradil and TH1177 inhibit ORAI channels, but other T-type Ca^2+^ channel inhibitors show no effect. This discrepancy suggested that the inhibitory action of TH1177 on ORAI channels is structure-specific to the compound, but irrelevant to the class inhibitory effect on T-type Ca^2+^ channels. To further elucidate the structure–activity relationship, we have examined the benzhydryl derivatives with high structural similarity to TH1177, such as clemastine, cloperastine, and diphenhydramine. Clemastine, with a pyrrolidine ring substructure, exhibits specific blocking activity for ORAI1 and ORAI2 isoforms, but both cloperastine and diphenhydramine, without the pyrrolidine ring substructure, only show small or no effects on ORAI channels, suggesting that the pyrrolidine ring is essential for channel-blocking activity. Clemastine is a first-generation histamine H1 receptor antagonist that is widely used for treating allergic disorders and pruritus and has also been investigated in multiple sclerosis [30]. Its newly identified activity against ORAI1 and ORAI2 could contribute to some of its immunomodulatory effects, although this possibility remains to be tested directly. The present findings provide a rationale for examining whether ORAI inhibition occurs at clinically achievable clemastine concentrations and whether this mechanism could support repurposing in ORAI-associated disorders. Such studies are needed in the future to distinguish ORAI-dependent effects from established H1 receptor antagonism and other known pharmacological actions of clemastine.

## 5. Conclusions

TH1177 is a pan-inhibitor for ORAI1-3, but is more potent on ORAI2, while clemastine is a specific inhibitor for ORAI1 and ORAI2. The action site for TH1177 is extracellularly located, which gives a new clue for further compound screening and future development of a potential anti-proliferative drug. The new pharmacological profile of clemastine may shed light on its repurposing as an immune regulator via inhibiting ORAI channels.

## Data Availability

The original contributions presented in this study are included in the article/Supplementary Materials. Further inquiries can be directed to the corresponding authors.

## Supplementary Materials

The following supporting information can be downloaded at: https://www.mdpi.com/article/10.3390/biom16091372/s1, Figure S1: Clemastine had no effect on ORAI3 current; Figure S2: Dose-response curve for clemastine on the inhibition of ORAI1 current (*n* = 4–8); Figure S3: Effects of TH1177 on Ca^2+^ release and SOCE in cells overexpressing ORAI2 or ORAI3; Figure S4: Effects of TH1177 on cell proliferation.

## Abbreviations

The following abbreviations are used in this manuscript:

2-APB	2-aminoethoxydiphenyl borate
CRAC	Ca^2+^-release activated calcium channel
DES	Diethylstilbestrol
ER	Endoplasmic reticulum
EYFP	Enhanced yellow fluorescent protein
HAEC	Human aortic endothelial cells
IV	Current-voltage relationship
LDH	Lactate dehydrogenase
SERCA	Sarco/endoplasmic reticulum calcium-transporting ATPase
SOCE	Store-operated calcium entry
STIM1	Stromal interaction molecule 1
TG	Thapsigargin
TRPC	Transient receptor potential canonical
TRPV	Transient receptor potential vanilloid
PI	propidium iodide
