# Supplementary material for: TH1177, Clemastine, and Benzhydryl Derivatives Act as Novel Extracellular Blockers of ORAI Channels
Source: Biomolecules. 2026 Sep 21;16(9):1372. doi: 10.3390/biom16091372 (PMC13604178; doi:10.3390/biom16091372)
Supplement: Supplementary file 1 [file biomolecules-16-01372-s001.zip › biomolecules-4491555-supplementary.pdf]

TH1177, Clemastine, and Benzhydryl Derivatives Act as Novel Extracellular Blockers of ORAI Channels

Pengyun Li [1,2], Hussein N. Rubaiy [1], Thomas Hallett [1], Yi Liang [2], Gui-Lan Chen [1,2], Bo Zeng [1,2], Pingping Zheng [1], Nawel Zaibi [1], Dong-Na Su [1], Andrew N Boa [3] and Shang-Zhong Xu [1,3,*]

Supplementary figures

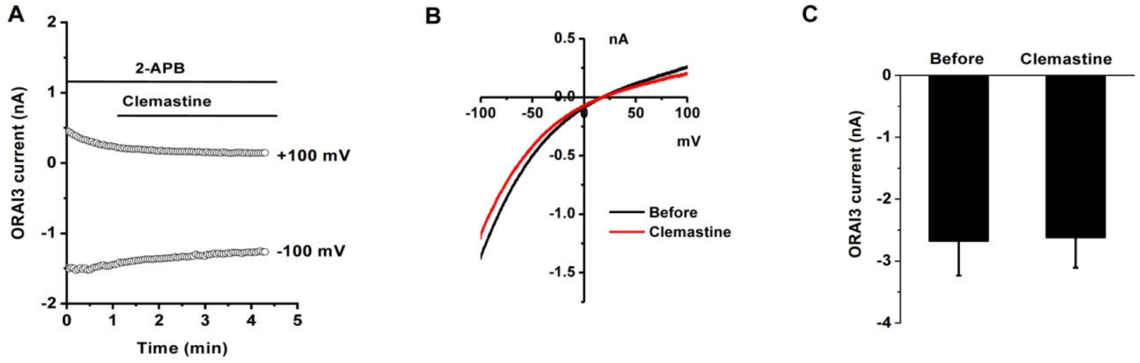

Supplementary Figure S1 Clemastine had no effect on ORAI3 current. (A) The cells overexpressing ORAI3 were perfused with divalent cation free solutions (DVF) and the current was induced by 2-APB (100 µM). Clemastine (100 µM) was added. (B) IV curve for (A). (C) Mean ± SEM data (*n* = 5).

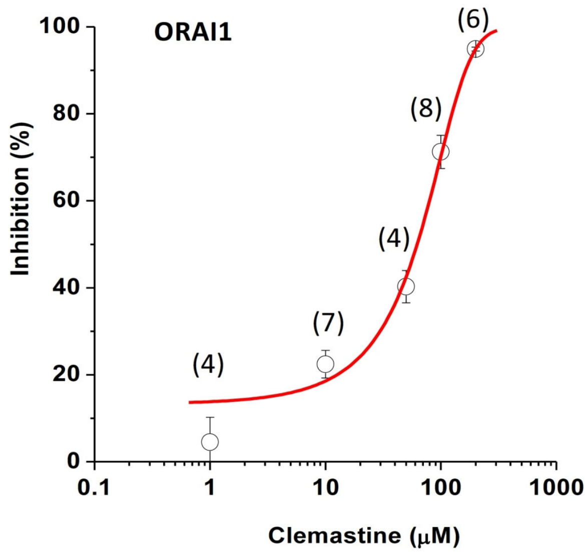

Supplementary Figure S2 Dose-response curve for clemastine on the inhibition of ORAI1 current (n = 4-8). The IC_{50} = 68.6±5.7 μM.

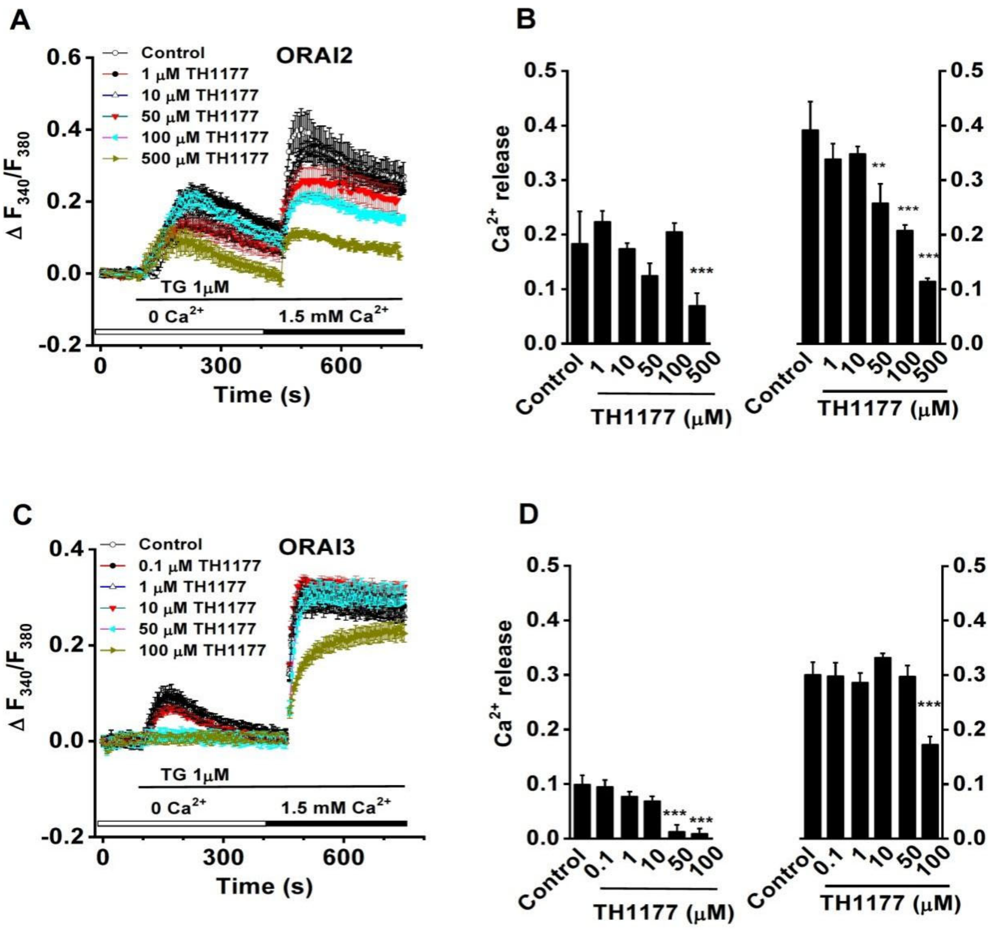

Supplementary Figure S3 Effects of TH1177 on Ca^{2+} release and SOCE in cells overexpressing ORAI2 or ORAI3. (A) HEK-293 T-REx overexpressing with ORAI2/STIM1 were loaded with Fura-PE3/AM and the ER Ca^{2+} release was induced by 1 μM thapsigargin (TG). The fluorescence intensity at a ratio of F340/380 was monitored in the groups with or without pretreatment with different concentrations of TH1177. The SOCE was evoked by refilling 1.5 mM Ca^{2+} in the bath solution. (B) Mean data was measured at the peak of Ca^{2+} release or peak of SOCE (n=8). (C) HEK-293 T-REx cells overexpressing with ORAI3/STIM1 and the effects of TH1177 on SOCE. (D) Mean data for peak Ca^{2+} release and SOCE (n = 8). **p< 0. 01, ***p < 0.001.

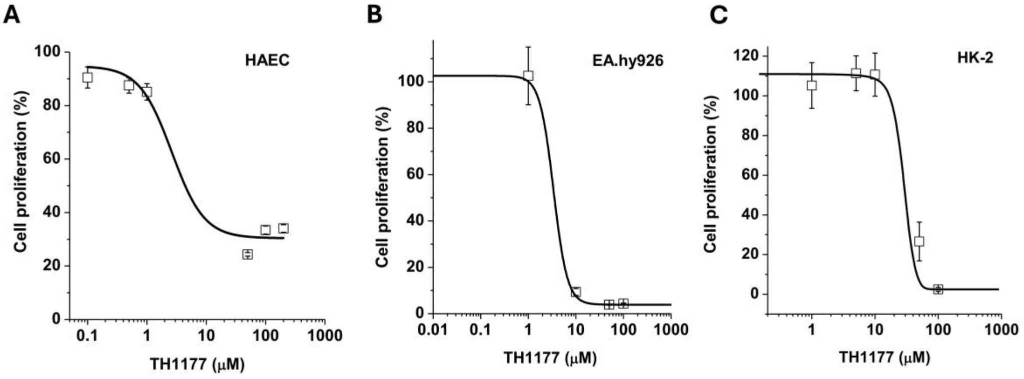

Supplementary Figure S4

Effects of TH1177 on cell proliferation. Cell proliferation was measured using WST-1 assay. Data were fitted with Boltzmann equation to generate the dose-response curves and IC50. (A). HAEC. (B). EA.hy926. (C). HK-2 cells.
